# Delayed mesh infection after inguinal hernia repair: a case report

**DOI:** 10.1093/jscr/rjab399

**Published:** 2021-09-22

**Authors:** Homare Ito, Kenji Matsumoto, Toshiaki Terauchi, Masaru Kimata, Alan Kawarai Lefor, Hiroharu Shinozaki

**Affiliations:** Department of Surgery, Saiseikai Utsunomiya Hospital, Utsunomiya, Tochigi, Japan; Department of Surgery, Division of Gastroenterological, General and Transplant Surgery, Jichi Medical University, Tochigi, Japan; Department of Surgery, Saiseikai Utsunomiya Hospital, Utsunomiya, Tochigi, Japan; Department of Surgery, Division of Gastroenterological, General and Transplant Surgery, Jichi Medical University, Tochigi, Japan; Department of Surgery, Saiseikai Utsunomiya Hospital, Utsunomiya, Tochigi, Japan; Department of Surgery, Saiseikai Utsunomiya Hospital, Utsunomiya, Tochigi, Japan; Department of Surgery, Division of Gastroenterological, General and Transplant Surgery, Jichi Medical University, Tochigi, Japan; Department of Surgery, Saiseikai Utsunomiya Hospital, Utsunomiya, Tochigi, Japan

## Abstract

Delayed deep mesh infection is a rare complication and the precise mechanism of its development is unknown. We report a case of delayed deep mesh infection after inguinal hernia repair. A 65-year-old man was admitted for treatment of colon cancer. He had a history of bilateral hernioplasty repaired with mesh-plugs 6 years previously. Fluorine-18 fluorodeoxyglucose positron emission tomographic scan showed positive findings in the right inguinal region similar to cancer. He had no complaints or findings to suspect mesh infection. Postoperative computed tomography scan over time revealed a fluid collection with inflammation. Eleven years after hernia repair, the patient presented with inflammation in the right inguinal region and emergency operation was performed. An abscess cavity was found and the mesh-plug covered with granulation tissue was removed. The patient remains free of recurrence of inguinal hernia or inflammatory changes after 3 years of follow-up.

## INTRODUCTION

Inguinal hernia repair is the most common elective procedure performed by general surgeons, usually with the use of prosthetic mesh. Mesh-related complications have been increasing in incidence [1, 2]. Postoperative mesh infection is rare, but a particularly troublesome complication occurring in ~5.0% of patients [3]. Late onset deep mesh infections account for <1% of infections, but have been reported to occur several years after surgery [4–8]. Various possible mechanisms for the establishment of late-onset deep mesh infection have been suggested [9, 10], but the details remain unclear. We report a patient with a deep mesh infection that developed 11 years after hernia repair for which serial imaging findings were obtained.

## CASE REPORT

**Figure 1:**
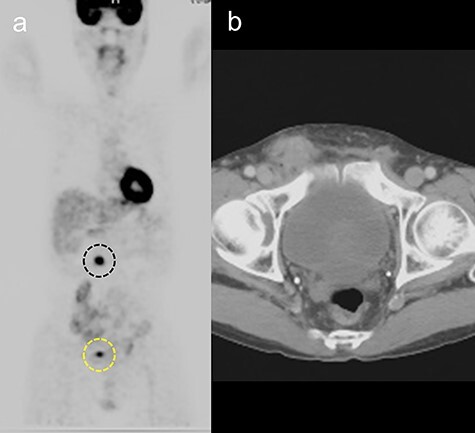
(**a**) Fluorine-18 fluorodeoxyglucose positron emission tomographic scan showing positive findings in the transverse colon (black dotted line) and right inguinal area (yellow dotted line). (**b**) Computed tomography scan showing a mass with indistinct margins in the right inguinal area, 5 years after hernia repair.

**Figure 2:**
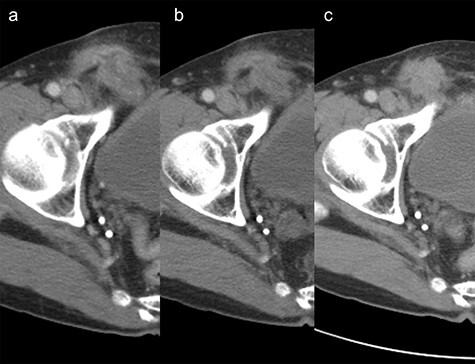
Change over time after transverse colon resection in the right inguinal area on computed tomography scan. (**a**) 7 years year after hernia repair. (**b**) 8 years after hernia repair. (**c**) nine and a half years after hernia repair.

A 65-year-old man was admitted for the treatment of transverse colon cancer. He had a history of bilateral hernioplasty with mesh-plugs 6 years previously at another hospital. He had no complaints or findings on physical examination referable to the hernia repair. Fluorine-18 fluorodeoxyglucose positron emission tomographic (PET) scan showed positive findings in the transverse colon and in the right inguinal region prior to colon resection (Fig. 1a). Computed tomographic (CT) scan revealed a mass with indistinct margins in the same area highlighted on the PET scan (Fig. 1b). Inguinal lymph node metastasis was suspected and ultrasound-guided fine-needle biopsy performed, which showed no evidence of malignancy. After transverse colon resection, he was followed up with CT scans performed every 6 months. For three and a half years after the colon resection (Nine and a half years after hernia repair), the right inguinal region showed progressive changes (Fig. 2a–c). At 1 year after colon resection (7 years after hernia repair), fluid retention was observed, but by the second year (8 years after hernia repair), it was slightly reduced. After three and a half years after colon resection (nine and a half years after hernia repair), the fluid had resorbed completely, similar to the findings at 6 years after hernia repair. The patient had no complaints referable to the right inguinal region during follow-up.

**Figure 3:**
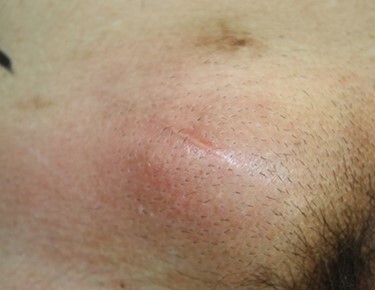
Physical findings included redness and swelling along the surgical scar.

**Figure 4:**
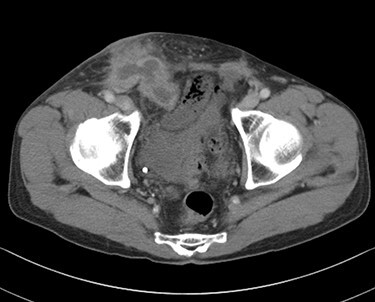
Computed tomography scan revealed abscess formation with contrast effect in the right inguinal area.

**Figure 5:**
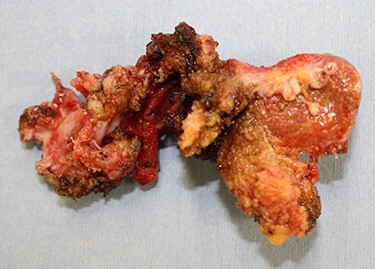
Resected mesh covered with granulation tissue.

Eleven years after hernia repair, the patient presented with redness, pain and swelling in the right inguinal region (Fig. 3). Laboratory studies revealed mild elevation of inflammatory markers (white blood cell count 13 000 /μl, C-reactive protein 2.8 mg/dl). Physical examination showed mild tenderness at the area of erythema. CT scan revealed abscess formation with contrast effect in the right inguinal region (Fig. 4). The patient was diagnosed with a mesh infection and abscess formation. Emergency operation was performed. The abscess cavity was opened and granulation tissue and the mesh-plug removed (Fig. 5). The aponeurosis of the external oblique muscle was repaired, with no reconstruction of the posterior wall. A closed drain was inserted subcutaneously at the time of wound closure. Abscess culture was positive for *Enterobacter cloacae*. He did well postoperatively except for a surgical site infection, discharged on postoperative Day 14. The patient remains free of recurrence of inguinal hernia after 3 years of follow-up.

## DISCUSSION

This report shows progressive changes on imaging studies during the development of a delayed deep mesh infection. This clinical history confirms two important points: (i) late-onset deep mesh infection may occur after a long period of persistent inflammation and (ii) no reinforcement of the posterior wall is required after removal of infected mesh.

Mesh-related infections after hernia repair are divided into early superficial infections and late-onset deep mesh infections, occurring in ~5% of patients overall [3]. The delayed deep mesh infection is extremely rare, with reported incidence rates of 0.09 to 0.35% [4–8]. The early superficial infection is usually due to operative contamination and may respond to non-operative treatment, whereas deep mesh infections have an unclear mechanism of development and require mesh removal. Microorganisms have been reported to be responsible for infection of the prosthetic mesh, which becomes a vascularized foreign body [9]. Bacteria typically involved in mesh infections include coagulase-negative Staphylococci, *Staphylococcus aureus*, and enteric gram-negative bacteria that have the capacity to form a biofilm [6, 8]. We detected *E. cloacae* in the abscess culture consistent with a previous report [8]. Darouiche *et al.* [11] reported that immediately after mesh insertion, granulation tissues with blood flow composed of host-derived adhesives (e.g. fibrinogen, fibronectin and collagen) form on the surface of the mesh, providing a scaffold for bacteria to attach and form a biofilm. However, late-onset deep mesh infections are very rare, and other factors leading to bacterial growth must be present to establish an infection. Mann *et al.* [10] suggested that late-onset prosthetic infections may arise as a complication of a persistent fluid collection. Supporting the above findings, although this patient had no right inguinal symptoms, CT scan showed persistent inflammation in the right inguinal area, fluid present. Given that water is essential for bacteria to grow, CT scan findings suggested that the right inguinal area was a likely site for bacterial growth.

Removal of infected prosthetic mesh makes it difficult to identify tissue planes intraoperatively due to the accompanying inflammatory response. Fawole *et al.* [7] reported that careful surgical dissection is necessary to prevent intraoperative damage to structures, and reinforcement of the transversus fascia is not required. In their follow-up patients, only 2/14 patients developed hernia recurrence, but these patients were asymptomatic and were treated non-operatively. Rehman *et al.* [12] reviewed six case series and a case report. In a total of 40 patients, only one required reoperation due to recurrence. We didn’t reinforce the posterior wall in the present patient because severe inflammation made accurate anatomic identification of the layers impossible. Wound closure after drainage of the abscess likely resulted in the superficial surgical site infection and should have been left open. The patient remains free of recurrence of inguinal hernia after 3 years of follow-up.

In conclusion, this report illustrates the development of a late-onset deep mesh infection with serial imaging studies. Deep mesh infection may occur after a persistent latent infection. Reinforcement of the posterior wall after removal of the infected mesh is not necessary.

## AUTHORS' CONTRIBUTIONS

H.I. carried out collection of patient’s data and drafted manuscript. K.M. helped to collect patient’s data. T.T., M.K., J.F. and H.S. checked manuscript. A.L. proofread the English language. All authors read and approved the final manuscript.

## FUNDING

The authors declare that they have no funding.

## CONSENT

Written informed consent was obtained from the patient for publication of this case report and accompanying images.

## CONFLICT OF INTEREST STATEMENT

None declared.
